# Influence of the Polymerization Modes on the Methacrylic Acid Release from Dental Light-Cured Materials—In Vitro Study

**DOI:** 10.3390/ma15248976

**Published:** 2022-12-15

**Authors:** Anna Lehmann, Kacper Nijakowski, Agnieszka Drożdżyńska, Martyna Przybylak, Patryk Woś, Anna Surdacka

**Affiliations:** 1 Department of Conservative Dentistry and Endodontics, Poznan University of Medical Sciences, 60-812 Poznan, Poland; 2 Department of Biotechnology and Food Microbiology, Poznan University of Life Sciences, 60-627 Poznan, Poland; 3 Student’s Scientific Group in Department of Conservative Dentistry and Endodontics, Poznan University of Medical Sciences, 60-812 Poznan, Poland

**Keywords:** dental material, restorative material, composite, resin-modified glass ionomer, pH, acidity, methacrylic acid, oral environment, dental filling, dental restoration

## Abstract

The study focuses on the problem of lowering the pH around a composite filling concerning the polymerization modes and methacrylic acid release, which may affect not only the oral health but also the whole organism. A total of 90 specimens (30 of each: Filtek Bulk Fill, Evetric and Riva LC) were placed in 90 sterile hermetic polyethene containers with saline and incubated at 37 °C. Ten samples of each material were light-cured for 40 s with one of the three curing modes: full power mode (FPM), ramping mode (RM) and pulse mode (PM). The pH and methacrylic acid release evaluation were performed at the following time points: after 2 h and after 3, 7, 21 and 42 days from the specimen preparation. Regardless of light-curing mode, all used materials were characterized by a gradual elevation in methacrylic acid concentration. Only for Filtek Bulk Fill, increased methacrylic acid release was closely associated with lower pH. The choice of the polymerization mode has no significant influence on the methacrylic acid release. However, further research about composite light-curing is necessary to create the procedure algorithm, reducing the local and systemic complications associated with composite fillings.

## 1. Introduction

Although the quality of dental composite materials has improved significantly since their introduction in the 1960s, the lifetime of these restorations is still much shorter than that of amalgams. It is about 6–7 years for small, single-surface filling and only about 4 years for multi-surface filling [1,2]. Degradation, discoloration and secondary caries are the main factors limiting the life of composites [3,4,5].

One of the main problems is the incomplete polymerization of the composite resins used in vivo. It has been shown that the conversion rate is around 60–75% [6,7]. Another problem with the use of composites is the inhibition of polymerization in surface layers exposed to oxygen. If not removed, during polishing, they will release monomers or degradation products related to the thickness of the unpolymerized layer. However, polishing removes the inhibition layer but generates roughness of the material and further complications in the form of plaque accumulation and secondary caries [8,9,10].

For good-quality composite materials, most of their monomers must be converted to polymers during the polymerization reaction. Hydrolytic degradation can lead to the release of various compounds, such as formaldehyde, benzoic acid, triethylene glycol and bishydroxypropoxyphenyl–propane [11,12,13]. It is suspected that many other unidentified compounds are degradation products of composite additives, i.e., inhibitors, catalysts, accelerators and UV stabilizers [14]. Previous studies have shown that, in particular, the dimethacrylate polymer exhibits significant amounts of unsaturated monomers in the final product [15,16,17]. Furthermore, di- and monomethacrylates hydrolyze to methacrylic acid and alcohols at neutral pH. Methacrylic acid is probably released from the degradation of dimethacrylates bound in the matrix with only one end of the molecule. In vitro studies with methacrylates have confirmed that their cytotoxicity is concentration-dependent, and they are toxic to osteoblasts and periodontal cells [18,19,20].

Devices for the polymerization of composite materials are divided into quartz–tungsten halogen (QTH), diode (light-emitting diode; LED), plasma (plasma arc cutting; PAC) and laser (argon lasers). LED lamps are considered the best in terms of both curing efficiency and heat generation. Researchers also indicate the need to establish operating modes tailored to specific commercial materials [21,22]. The spectral output of the dental clinic LEDs consists of the absorption peak of camphorquinone (CQ; 400–500 nm, peak at 470 nm), the most used photoinitiator in resin composites. The conversion rate is the number of carbon double bonds (C=C) present in the monomers, which are converted into single bonds (C–C) during the polymerization process, forming polymer chains, thus reducing the risk of the release of toxic monomers. The literature analysis shows that the most important factors for increasing the conversion are the exposure time, the shortest possible distance from the material surface and the reduction of oxygen inhibition [23,24].

LED curing lights offer clinicians several working modes but no indication of which mode to use in a particular situation. According to the literature, the best mode for dental composite is the so-called soft start. Its use for irradiation provides the relatively highest percentage of polymerization and the lowest shrinkage. This is due to the slow formation of several polymerization nuclei and a better cross-linking of the polymer [25,26,27].

However, it should be emphasized that the data on the release of substances from resin-based materials after different light-curing modes is poor. There is reliable information regarding the biological interactions between resin components and various tissues. Some microbial interaction is suspected, which may indirectly contribute to secondary caries and pulp irritation [28]. The allergenic effect of methacrylates, especially on medical personnel, is also essential [29]. That is why it is so important for patients and professionals that the biological effects of using resin fillers are clarified in the near future [30,31].

On the other hand, glass ionomer cements (GICs) are the composite product of the acid–base reaction between an aqueous poly(acrylic) acid solution and a calcium–fluoro-alumino–silicate glass powder, to form silica and poly–salt hydrogels with unreacted glass particles [32]. Resin-modified glass ionomer cement set via an acid–base reaction and light-activated free radical polymerization. The main composition of the powder phase is fluoroaluminosilicate glass. The liquid phase contains a copolymer of polyacrylic acids, a resin monomer, such as 2-hydroxyethyl methacrylate (HEMA) and photoinitiator [33].

Too shallow depth of cure and the need to apply multiple layers of the composite, which posed the risk of air bubbles or moisture contamination, led to the introduction of bulk-fill composites to the market. The unique advantage of this new material is that it can be placed in 4 mm layers [7]. Manufacturers mentioned that the main advancement of bulk-fill composite materials, namely increased depth of cure, higher translucency and low polymerization shrinkage, are related to modifications in the filler content and organic matrix [7,34,35]. Research shows, however, that the conversion rate of bulk fill composites fluctuates between 40–60%, which is similar to conventional composites [7,36,37]. This indicator in the case of resin-modified glass ionomer cements is about 50–65% [38]; however, various chemical additives can significantly increase this percentage [39]. Our previous study showed the high acidifying potential of bulk–fill material [9]. Will this be reflected in the amount of methacrylic acid released?

It is challenging to find in the literature a detailed analysis of the pH change of dental materials concerning the release of methacrylic acid and the polymerization mode. As already mentioned, the direct lowering of the pH of the filling adjacent to the tooth tissues and the cytotoxic effect of methacrylic acid are direct etiological factors of caries and may affect the prognosis, durability of the filling and the condition of the pulp [40]. There is no answer to whether composite degradation by-products, at low concentrations detected in vitro, may show persistent cytotoxic effects in vivo [11,12]. Perhaps establishing reference ranges of released monomers will help improve dental composite material in the future.

Our study aimed to assess the pH of the composite material and the amount of released methacrylic acid depending on the polymerization mode. We made the following hypotheses:

1. Acidification around the filling correlates with the amount of methacrylic acid released.
2. Polymerization mode affects the amount of methacrylic acid released.
3. Selected materials acidify the environment more after the selected polymerization mode.

## 2. Materials and Methods

### 2.1. Materials Used in the Study

In this in vitro study, we used three dental restorative materials. Detailed characteristics of the materials are presented in Table 1.

### 2.2. Specimen Preparation

All specimen preparation was done by one operator to reduce variability. Ninety specimens (30 from each material) were prepared using metal moulds with 6-mm diameter and 2-mm thickness. All materials were inserted into the mould and intentionally overfilled. Then the mould was sandwiched between transparent Mylar strips to expel excess material. Samples of each material (*n* = 10) were light-cured directly at the surface for 40 s with one of the three curing modes, according to the manufacturer’s instructions (Woodpecker LED.H, Guilin Woodpecker Medical Instrument Co., Guangxi, China). Light-cure modes used in the study:

- Full power mode (FPM), LED works in full power, 1000 mW/cm^2^
- Ramping mode (RM), LED turns from weak to stronger and reaches the highest power in 5 s
- Pulse mode (PM), LED pulsating between 850 mW/cm^2^–1000 mW/m^2^ in 2 s intervals.

### 2.3. Specimen Storage and pH Evaluation

The samples were placed in 90 sterile hermetic containers and incubated at 37 °C. To each container, 5 mL of saline (0.9% NaCl solution; Polpharma, Stargard Gdanski, Poland; lot: 1280620) were added.

The pH of the saline solution from each sample was measured using Halo HI13302 (Hanna Instruments, Olsztyn, Poland). Between procedures, the electrode was cleaned and recalibrated. All measurements were made in duplicate and then averaged. The pH evaluation was performed at the following timepoints: after 2 h and after 3, 7, 21 and 42 days from the specimen preparation.

### 2.4. High Performance Liquid Chromatography Analysis

Determinations of methacrylic acid were carried out on Agilent 1260 Infinity II system consisting of autosampler (model G7129A), pump (model G7111A) and DAD (model G7115A) set at 210 nm. Analysis was performed isocratically at a flow rate 0.8 mL/min, at 40 °C, on column Rezex ROA-Organic Acid H+, 300 × 7.8 mm (Phenomenex). 10 mM sulphuric acid as a mobile phase was used. Samples were filtered (0.22 µm, Millex-GS, Millipore, Burlington, MA, USA), and the injection volume was set at 20 µL. Standard was used to identify peaks in chromatograms, and peak area was used to determine the concentrations of the samples. It was done by computer integration operated in the mode of external standards.

### 2.5. Statistical Analysis

The determined pH values and methacrylic acid concentrations were analyzed using a two-way repeated-measures analysis of variance (in the model with the interaction). The variables were compared separately at specific points in time depending on the kind of used dental restorative material and polymerization mode. The significance level was defined as α = 0.05. The statistical analysis was performed using Statistica 13.3 (Statsoft, Cracow, Poland).

## 3. Results

**Figure 1:**
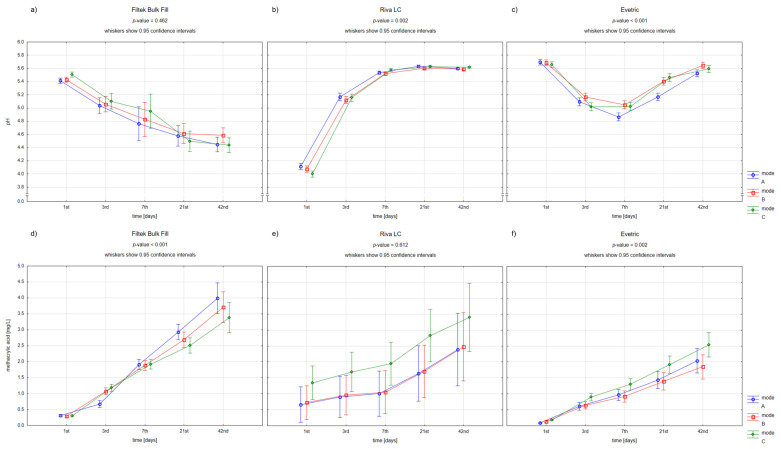
Repeated-measures analysis of variance for pH values (**a**–**c**) and methacrylate concentrations (**d**–**f**) in the individual timepoints for the used dental restorative materials depending on the polymerization mode (A—full power mode, B—ramping mode, C—pulse mode).

**Figure 2:**
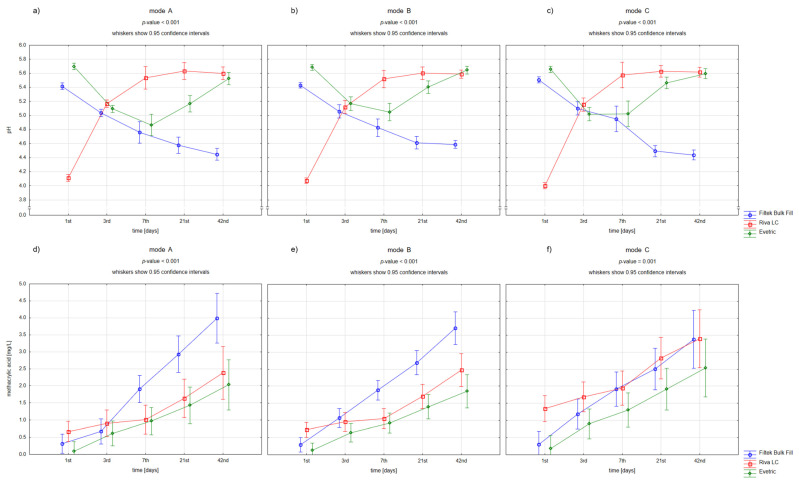
Repeated-measures analysis of variance for pH values (**a**–**c**) and methacrylic acid concentrations (**d**–**f**) in the individual timepoints for the used polymerization modes depending on the dental restorative material (A—full power mode, B—ramping mode, C—pulse mode).

Figure 1 and Figure 2 present the differences in pH changes (a–c) and in methacrylic acid levels (d–f) over time depending on the used dental materials and polymerization modes.

For all used materials, the pH values were not dependent on the kind of polymerization mode. Riva LC had the lowest baseline pH, followed by a significant pH increase to baseline values for both composite materials. In contrast, Filtek Bulk Fill showed a decrease in pH of about 1 unit over 42 days. Interestingly, for Evetric samples, the pH values decreased in the first week and then increased to the baseline levels.

Moreover, all used materials were characterized by a gradual elevation in methacrylic acid concentration. The largest increase was observed in Filtek Bulk Fill samples, which also reached the highest level after 42 days. In turn, the highest baseline methacrylic acid release had Riva LC. There was a difference between the polymerization modes on day 3 of the study for Filtek Bulk Fill and Evetric—the first samples in mode A (full power mode) demonstrated significantly lower methacrylic acid concentrations and the second samples in mode C (pulse mode) higher than the others.

Only for Filtek Bulk Fill samples, the clear inverse relationship between changes in pH and methacrylic acid levels was found. Increased methacrylic acid release was closely associated with lower pH.

## 4. Discussion

The majority of resin degradation studies have been done in water to mimic oral conditions. Water diffuses into the composite and accumulates between the resin and filler material, reacting with the silane coupler and filler material to release degradation products into the solution [41,42]. Various methods are used to identify residual monomers and degradation products. Methacrylates, such as Bis–GMA, UDMA or Bis–EMA, as large molecular size monomers, are well detected by high-performance liquid chromatography (HPLC) or HPLC mass spectrometry (HPLC/MS).

It is known that the pH constantly changes in the oral cavity, so our model tested in a neutral environment has some limitations. The consumption of food, drinks, hygiene products, medications and even the daily routine cause changes in the environment in the oral cavity [43,44]. Creating a perfect in vitro research model is a challenge for researchers.

Composite fillings are exposed to various conditions in the oral cavity, such as the mentioned changes in pH and ambient temperature. However, hydrolytic degradation is still one of the more difficult challenges [45]. But where do the methacrylates in the hydrolyzate come from? Dental composites are based on dimethacrylates such as bisphenol A glycol dimethacrylate (Bis–GMA), ethoxylated bisphenol A dimethacrylate (Bis–EMA), urethane dimethacrylate (UDMA), triethylene glycol dimethacrylate (TEGDMA). They contain a number of hydrolyzable bonds, such as esters, ethers, urethanes and amides, that can be cleaved by hydrolysis [11]. However, hydrolysis and biodegradation in composites are not limited to the polymer matrix. The interface between the organic polymer and the inorganic filler is also susceptible to chemical attack. The silane coupling agents used are amphiphilic and contain two different types of functional groups. One is methacrylate, which provides adhesion to the polymer matrix through polymerization. The second is a silicon functional group (alcoxy) that can react with the active sites of inorganic fillers. The methacrylate ester and siloxane bonds tend to break down in a humid environment, accelerating the degradation of the composite [46,47].

In our study, methacrylic acid was noted in all samples. All tested materials were characterized by a gradual increase in methacrylate concentration. Numerous studies have analyzed [13,14,29,36] how long methacrylic acid can leach out of the composite. We asked the question of which material will release the most of it. The highest amount of released methacrylic acid was determined in samples from bulk-fill material. Our results contradict the common opinion that UDMA resin is more stable than other resins used in dental composites [16,41,48]. Also, for the Filtek Bulk Fill samples, there was a clear inverse relationship between pH changes and methacrylate levels. Increased methacrylate release was closely related to lower pH. In our study, Evetric composite, containing Bis–EMA resin, turned out to be the least acidifying. Our results correspond with Sideridou et al. in this respect [41]. It has been shown that in the Bis–GMA/TEGDMA mixtures, a greater amount of free monomer reacts. It is believed that the bulky and rigid Bis–GMA, which has a strong ability to form hydrogen bonds through its hydroxyl groups, is leached out much more slowly than the small and flexible TEGDMA. The lower solubility of nano-hybrids is also supported by the fact that, in this type of composite, we are dealing with an enlarged filler–matrix interface and a reduced filler size [13,49,50]. In turn, the highest initial release of methacrylate and the lowest initial pH were recorded with Riva LC. It was followed by a significant increase in pH to values known for conventional composite materials. This probably proves the effect of the high content of polyacrylic and tartaric acids on the initial pH. Gradual, slow polymerization (dark phase) can deactivate these acids and contribute to the pH increase [38].

It is well-known that the higher the energy density delivered during light curing, the greater the degree of conversion and polymerization shrinkage [51]. In our study, for all the materials used, the pH values were not dependent on the type of polymerization mode. However, we have seen changes regarding methacrylic acid. There was a difference between the polymerization patterns on the third day of the study for Filtek Bulk Fill and Evetric. The first composite in full power mode showed significantly lower concentrations of methacrylic acid, and the second in pulse mode higher than the others. Also, Riva LC in pulse mode released the most methacrylic acid. The pulse mode (mode C) seemed to be the least favorable, resulting in higher concentrations of methacrylic acid. It is believed that a small number of free radicals are produced during pulsed irradiation, and the conversion of double bonds is slower. Proponents of this method report that a lower initial conversion can result in polymer chains with greater mobility, which consequently allows for better curing [52]. Does this mean that when using the pulse mode (mode C), the exposure time should be significantly extended? The answer to this question requires further analysis.

The standard polymerization mode (mode A) seemed to be the worst for the bulk-fill composite in our study. This may be due to high initial energy and lack of time for the so-called dark phase of polymerization. However, the popular method of soft start polymerization, which clinicians like, seems to be still valid. The initial, low power density results in polymers with a similar degree of curing as in the conventional continuous technique. It has been hypothesized that the slow polymerization is probably related to the relatively small number of polymer growth centers generating a small number of free radicals, which results in a more linear polymer structure with lower cross-link density, delayed gel point and lower stress shrinkage while maintaining a constant degree of cure and mechanical properties [52,53,54].

Dental composite materials are still being thoroughly tested regarding biocompatibility, adhesion to the tooth or various strength parameters. Until there are clear guidelines on clinical management, it is worth remembering that the best composite properties so far can be obtained with indirect works. Advantages of this technique include enhanced physical and mechanical properties afforded by the extraoral tempering process because of the increased conversion degree, greater operator control over the final marginal adaptation, surface finishing, polishing and anatomy of the restoration [55]. However, such effort is not possible for every patient due to the high costs and time needed to work in the laboratory.

## 5. Conclusions

In our study, acidification correlated with the amount of methacrylic acid, especially clearly in the case of the bulk–fill composite. Among the polymerization modes, the pulse mode seemed to be the least favorable. Also, the pH values were not dependent on the polymerization mode for all the materials used. Further research on the polymerization conditions is requisite for better identifying the factors promoting the methacrylic acid release.

## Figures and Tables

**Table 1:** Detailed characteristics of the dental restorative materials used in the study.

Material	Material Group	Manufacturer	Acronym	Composition	Lot Number
Filtek Bulk Fill, Shade A3	Composite	3M/ESPE, Seedfeld, Germany	FBF	Organic matrix: AUDMA, UDMA, 1,12-dodecane-DMA 20 nm silica; Filler fraction (wt%/vol%) 76.5/58.4, Fillers: 4–11 nm zirconia, ytterbium trifluoride filler consisting of agglomerate 100 nm particles	N867070
Evetric, Shade A3	Composite	Ivoclar Vivadent, Schaan, Liechtenstein	ER	Organic matrix: Bis–GMA, Bis–EMA, UDMA; Filler fraction (wt%/vol%) 80–81/55–57, Fillers: barium glass, ytterbium trifluoride, mixed oxide, copolymers (size 40–3000 nm)	Y20235
Riva LC, Shade A3	resin-modified glass ionomer cement	SDI Limited, Victoria, Australia	RLC	Liquid: polyacrylic acid 20–30%, tartaric acid 5–10%, HEMA 20–25%, dimethacrylic acid cross linker 10–25%, acid monomer 10–20% Powder: fluoroaminosilicate powder 95–100%	J2011171

## Data Availability

Data are available on request from the corresponding author.
